# Chloroviruses Have a Sweet Tooth

**DOI:** 10.3390/v9040088

**Published:** 2017-04-22

**Authors:** James L. Van Etten, Irina Agarkova, David D. Dunigan, Michela Tonetti, Cristina De Castro, Garry A. Duncan

**Affiliations:** 1 Department of Plant Pathology and Nebraska Center for Virology, University of Nebraska-Lincoln, Lincoln, NE 68583-0900, USA; irina@unl.edu (I.A.); ddunigan2@unl.edu (D.D.D.); 2 Department of Experimental Medicine and Center of Excellence for Biomedical Research, University of Genova Viale Benedetto XV/1, 16132 Genova, Italy; tonetti@unige.it; 3 Department of Agricultural Sciences, University of Napoli, Via Università 100, 80055 Portici, NA, Italy; decastro@unina.it; 4 Department of Biology, Nebraska Wesleyan University, Lincoln, NE 68504-2796, USA; gduncan@nebrwesleyan.edu

**Keywords:** chloroviruses, giant viruses, hyaluronan synthesis, chitin synthesis, nucleotide sugar synthesis, glycan synthesis, glycan structures, glycoproteins

## Abstract

Chloroviruses are large double-stranded DNA (dsDNA) viruses that infect certain isolates of chlorella-like green algae. They contain up to approximately 400 protein-encoding genes and 16 transfer RNA (tRNA) genes. This review summarizes the unexpected finding that many of the chlorovirus genes encode proteins involved in manipulating carbohydrates. These include enzymes involved in making extracellular polysaccharides, such as hyaluronan and chitin, enzymes that make nucleotide sugars, such as GDP-l-fucose and GDP-d-rhamnose and enzymes involved in the synthesis of glycans attached to the virus major capsid proteins. This latter process differs from that of all other glycoprotein containing viruses that traditionally use the host endoplasmic reticulum and Golgi machinery to synthesize and transfer the glycans.

## 1. Introduction

In discussing enzymes involved in manipulating carbohydrates, one usually does not consider viruses to play a role in this important subject. However, as described in this review, chloroviruses (family *Phycodnaviridae*) that infect certain isolates of single-celled, eukaryotic chlorella-like green algae are an exception to this process because they encode enzymes involved in making extracellular polysaccharides, nucleotide sugars and the synthesis of glycans attached to their major capsid glycoproteins.

The plaque-forming chloroviruses are large icosahedral (190 nm in diameter), double-stranded DNA (dsDNA)-containing viruses (genomes of 290 to 370 kb) with an internal lipid membrane. They exist in inland waters throughout the world with titers occasionally reaching thousands of plaque-forming units (PFU) per mL of indigenous water. Known chlorovirus hosts, which are normally endosymbionts and are often referred to as zoochlorellae [1,2], are associated either with the protozoan *Paramecium bursaria*, the coelenterate *Hydra viridis* or the heliozoan *Acanthocystis turfacea* [3,4,5,6]. Four such zoochlorellae and their viruses are *Chlorella variabilis* NC64A and its viruses (referred to as NC64A viruses), *Chlorella variabilis* Syngen 2–3 and its viruses (referred to as Osy viruses), *Chlorella heliozoae* SAG 3.83 and its viruses (referred to as SAG viruses) and *Micractinium conductrix* Pbi and its viruses (referred to as Pbi viruses). The zoochlorellae are resistant to virus infection when they are in their symbiotic relationship, because the viruses have no way of reaching their hosts.

The genomes of 43 chloroviruses infecting these four hosts have been sequenced, assembled and annotated [6,7,8,9,10,11]. Collectively, the viruses encode genes from 643 predicted protein families; however, any given chlorovirus only has 330 to 416 protein-encoding genes (PEGs). Thus, the genetic diversity among these viruses is large, and many of the proteins are unexpected for a virus. With the exception of homologs solely in other chlorovirus members, about 50% of their PEGs do not match anything in the databases.

The prototype chlorovirus *Paramecium bursaria* chlorella virus type 1 (PBCV-1) is an NC64A virus [12]. PBCV-1 is an icosahedron (190 nm in diameter) with a spike-like structure at one vertex and a few external fibers that extend from some of the viral capsomeres [5,13]. The outer capsid layer covers a single lipid bilayered membrane, which is essential for infection. The PBCV-1 major capsid protein (named Vp54) is a glycoprotein, and three Vp54s form a trimeric capsomere, which has pseudo-six-fold symmetry. A proteomic analysis of PBCV-1 virions revealed that the virus contains 148 virus-encoded proteins and at least one host-encoded protein [10]. The PBCV-1 genome is a linear ~331-kb, non-permuted dsDNA molecule with covalently-closed hairpin termini. Identical ~2.2-kb inverted repeats flank each 35-nucleotide-long, incompletely base-paired, covalently closed hairpin loop [14,15]. The remainder of the PBCV-1 genome contains primarily single-copy DNA that encodes ~416 putative proteins and 11 transfer RNAs (tRNAs) [5]. The G + C content of the PBCV-1 genome is 40%; in contrast, its host nuclear genome is 67% G + C. PBCV-1 and other chlorovirus genomes contain methylated bases, which occur in specific DNA sequences. The methylated bases are part of chlorovirus-encoded DNA restriction and modification systems [16].

As the title of this review indicates, many of the chlorovirus genes encode enzymes involved in various aspects of carbohydrate metabolism. We have listed putative chlorovirus genes involved in carbohydrate metabolism, which are encoded by the 43 chloroviruses whose genomes have been sequenced, in Table 1, Table 2 and Table 4. Recombinant proteins have been produced from some of these genes, and the proteins have been characterized (indicated in bold in the tables). When some of the genes were initially cloned and the recombinant proteins characterized, the genes were hybridized to many other chlorovirus genomes by dot blots to determine the distribution of the genes. Because of the large number of viruses, these experimental results are not included in the tables, unless the virus genome was subsequently sequenced.

## 2. Chlorovirus Encoded Polysaccharide Synthesizing Enzymes

Three PBCV-1 encoded enzymes are involved in the synthesis of the extracellular matrix polysaccharide hyaluronan (also referred to as hyaluronic acid), including glycosyltransferase Class I hyaluronan synthase (HAS; Table 1) [17,18]. Until the *has* gene (*a098r*) was discovered in PBCV-1, hyaluronan was only thought to occur in vertebrates and a few pathogenic bacteria, where it forms an extracellular capsule, presumably to avoid the immune system [19,20]. Hyaluronan is an essential constituent of the extracellular matrix in vertebrates and consists of ~10,000 or more alternating β-1,4-glucuronic acid (GlcA) and β-1,3-*N*-acetylglucosamine (GlcNAc) residues. Typically, the HAS enzyme is located on the inner surface of the plasma membrane. The newly-synthesized hyaluronan then moves through the membrane and cell wall to the extracellular matrix.

**Figure 1:**
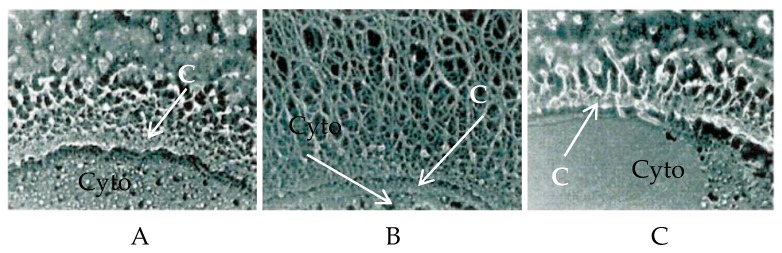
Location of hyaluronan on the surface of infected *Chlorella variabilis* NC64A cells and ultrastructural changes in the algal cell wall after chlorovirus *Paramecium bursaria* chlorella virus type 1 (PBCV-1) infection. The figure shows the cross-sections of (**A**) the surface of the uninfected cells; (**B**) cells at 4 h post-infection (PI); and (**C**) cells at 4 h PI that were treated with hyaluronan lyase. Note that after treatment with hyaluronan lyase, the cell surface resembles the surface of uninfected cells. C is the cell wall, and Cyto is cytoplasm. Micrographs were taken from Graves et al. [18] with permission.

PBCV-1 also encodes two enzymes involved in the biosynthesis of hyaluronan precursors, glutamine:fructose-6-phosphate amidotransferase (GFAT, gene *a100r*) and UDP-glucose dehydrogenase (UDP-GlcDH, gene *a609l*; Table 2) [21]. All three PBCV-1 genes involved in hyaluronan synthesis are expressed early during virus infection, and all three transcripts decrease significantly by 60 min post-infection (PI) [18,21]. However, these three genes do not function like an operon, although two of the genes, *a98r* and *a100r*, are adjacent to one another and are co-linear in the PBCV-1 genome. In contrast, *a609l* is located ~240 kb away and is transcribed in the opposite orientation [17]. The identification of these three genes led to the discovery that hyaluronan lyase-sensitive hair-like fibers begin to accumulate on the surface of PBCV-1-infected host cells by 15 min PI. By 4 h PI, the infected cells are covered with a dense fibrous hyaluronan network (Figure 1) [18].

Three additional enzymes are needed to convert glucosamine-6-phosphate (GlcN-6P) to UDP-*N*-acetylglucosamine (UDP-GlcNAc), and these enzymes (EC2.3.1.4, EC5.4.2.3, EC2.7.7.23) are encoded by the host [22]. This is not surprising because the host NC64A cell wall is predicted to contain chitin, which is a polymer of GlcNAc residues, and so, the alga must encode these enzymes. 

The *has* gene that encodes hyaluronan synthase is present in 12 of the 43 chloroviruses isolated from diverse geographical regions, including 5 NC64A viruses, 6 Pbi viruses and 1 Osy virus (Table 1). In contrast, the *udp-glcdh* gene is present in 40 of the 43 viruses, 14 of which have two copies of the gene, while the *gfat* gene is present in 27 chloroviruses, including 11 of the 14 NC64A viruses, all 14 Pbi viruses, one of the 13 SAG viruses and the only Osy virus that has been sequenced (Table 2). Both of these latter two genes are present in all of the 12 viruses that have a *has* gene, except for the one Osy virus that lacks a *udp-glcdh* gene.

Surprisingly, 19 of the 31 chloroviruses that lack a *has* gene have a gene encoding a chitin synthase (CHS). Chitin, an insoluble linear homopolymer of β-1,4-linked-GlcNAc residues, is a common component of insect exoskeletons, shells of crustaceans and fungal cell walls [23]. Chitin is rare in algal cell walls, although it has been reported to exist in some green algae [24]. Like the *has* gene, the *chs* gene is expressed as early as 10 min PI and peaks at 20–40 min PI, and the transcript disappears at 120–180 min PI. Furthermore, cells infected with *chs*-containing viruses produced chitin fibers on the external surface of their hosts [25]. As discussed below, many of the chloroviruses also encode chitinases and chitosanases.

At least one chlorovirus, CVK2, has replaced the PBCV-1 *has* gene with a 5-kb region containing *chs*, *udp-gdh2* (a gene encoding a second UDP-GlcDH) and two other ORFs [26]. Therefore, at least some chloroviruses have changed from HAS viruses to CHS viruses or vice versa, by swapping genes.

Two NC64A chloroviruses have both *has* and *chs* genes, and at least one of them forms both hyaluronan and chitin on the surface of their infected cells [25,27]. Finally, 12 chloroviruses lack both genes, and no extracellular polysaccharides are formed on the surface of cells infected with at least one of these viruses [18]. The fact that many chloroviruses encode enzymes involved in extracellular polysaccharide biosynthesis suggests that the polysaccharides, which require a large expenditure of ATP for their synthesis, are important in the virus life cycles. However, the extracellular hyaluronan does not play an obvious role in the interaction between PBCV-1 and its algal host because neither plaque size nor plaque numbers were altered by including either hyaluronidase or free hyaluronan in the top agar of the PBCV-1 plaque assay [17].

The three genes involved in synthesizing hyaluronan have probably been obtained rather recently in evolutionary terms because the coding portions of the PBCV-1 *gfat* and *udp-glcnc* genes are 44% G + C, while the *has* gene is 46.7% G + C. In contrast, PBCV-1, as well as all the NC64A viruses, have a 40% G + C content [11,21].

Currently, it is not known how or why the chloroviruses acquired these polysaccharide-synthesizing genes. We have considered the following possible evolutionary advantages for acquiring these genes: (1) the polysaccharides prevent infection by a second chlorovirus; (2) they cause the infected cells to clump with uninfected host cells, thus increasing the probability that progeny viruses can infect healthy host cells; (3) they prevent paramecia from taking up infected algal cells, (4) the chloroviruses have another host in nature, and this other host is attracted to or binds to hyaluronan or chitin on virus-infected algae, which would facilitate progeny-virus infections; or (5) polysaccharides increase the functional diameter of the infected cell, which might facilitate consumption by a predator. This could aid virus movement in the water column. In regards to the first possibility, it is known that attachment of other viruses to PBCV-1-infected cells at 4 h PI is inhibited when the external surface of the host is covered with hyaluronan fibers [18]. However, this is unlikely to be the explanation for the presence of hyaluronan because normally the host, *C. variabilis* NC64A, is only infected by one virus, and this restriction occurs in the first few min PI [28,29]. In regards to the second possibility, host cells often clump shortly after infection, and this phenomenon, which does not always occur, could be due to hyaluronan production. The last three possibilities have not been explored experimentally.

We have experimentally tried to address the question: does the presence of hyaluronan and/or chitin on the exterior surface of the host cell wall confer an evolutionary advantage to a virus that has one or both of these genes? To answer the question, chlorella cells were co-infected with combinations of chloroviruses that: (1) have both genes; (2) only have the *has* gene; (3) only have the *chs* gene; and (4) lack both genes. The resulting lysates were then added to fresh cells and allowed to replicate and lyse. After five passages, progeny viruses were plaqued, and 20 plaques were randomly picked to determine if one virus type dominated. However, after repeating these experiments several times, no consistent pattern was obtained [30].

To ideally conduct this experiment, one would like to either add the *chs* gene to the PBCV-1 genome so that both genes are present, replace the *has* gene with the *chs* gene or remove the *has* gene so that PBCV-1 lacked both genes. Unfortunately, this experimental protocol is currently not possible because procedures are not available for reverse genetic manipulation of chlorovirus genomes. Therefore, in the experiments described above, viruses were selected that had the desired properties and also had similar growth kinetics as PBCV-1.

In addition to not knowing why the chloroviruses acquired the *has* and *chs* genes, another question is: how are the newly-forming hyaluronan and/or chitin fibers moved through the membrane and the complex cell wall to the exterior of the algal host from the plasma membrane? This phenomenon would appear to be equivalent to pushing a thread through a furnace filter. One would expect the polysaccharide fibers to bunch up underneath the cell wall. In fact, this happened when the viral *has* gene was expressed in cultured tobacco cells [31]. Could a pilot protein(s) that is attached to the leading end of the polymer guide the hyaluronan chain through the wall?

## 3. Chlorovirus Encoded Nucleotide Sugar Metabolism Enzymes

**Figure 2:**
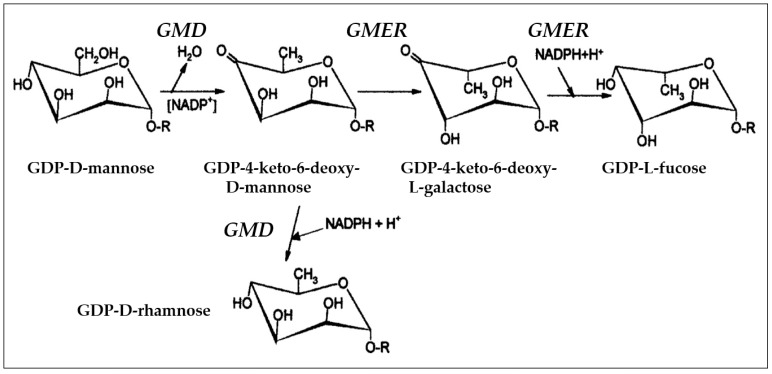
Scheme of the biosynthesis of GDP-l-fucose and GDP-d-rhamnose. PBCV-1 GDP-d-mannose 4,6 dehydratase (GMD) catalyzes both the dehydration of GDP-d-mannose to the intermediate GDP-4-keto-6-deoxy-d-mannose and the NADPH-dependent reduction of this latter compound to GDP-d-rhamnose. NADP^+^ serves as the cofactor for GMD during the internal oxidoreduction reaction involved in the dehydration process. The epimerization and the NADPH-dependent reduction of the 4-keto group leading to GDP-l-fucose are carried out by PBCV-1 GDP-4-keto-6-deoxy-D-mannose epimerase reductase (GMER). Figure was taken from Tonetti et al. [32] with permission.

Many chloroviruses also encode enzymes involved in nucleotide sugar metabolism, as well as other sugar metabolic enzymes (Table 2). Two enzymes encoded by all of the NC64A, SAG and Syn chloroviruses, GDP-D-mannose 4,6 dehydratase (GMD) and GDP-4-keto-6-deoxy-d-mannose epimerase reductase (GMER) (Table 2), comprise a highly-conserved pathway in bacteria, plants and animals that converts GDP-d-mannose to GDP-l-fucose (Figure 2) [32]. Fucose is found in glycoconjugates of many organisms, where it often plays a fundamental role in cell-cell adhesion and recognition [33]. The Pbi chloroviruses lack both *gmd* and *gmer* genes (Table 2) even though the glycans attached to the major capsid protein from the three evaluated Pbi viruses have fucose [34,35].

In vitro reconstruction of the pathway using recombinant PBCV-1 GMD and GMER proteins resulted in the synthesis of GDP-l-fucose as expected. Unexpectedly, however, the PBCV-1 GMD also catalyzed the NADPH-dependent reduction of the intermediate GDP-4-keto-6-deoxy-d-mannose, to form GDP-d-rhamnose. That is, the enzyme has two activities, and both sugars are produced in the infected cell [32]. The PBCV-1 recombinant GMD has another property that is unusual. Unlike recombinant GMDs from many other organisms, the viral encoded enzyme is very stable when stored at either 4 °C or −20 °C [32]. The PBCV-1 GMD enzyme was crystalized, and the structure resembles other GMDs [36].

A recombinant GMD protein encoded by another chlorovirus, *Acanthocystis turfacea* chlorella virus 1 (ATCV-1), which has 53% amino acid identity with the PBCV-1 GMD, was also characterized because the amino acid differences between the two enzymes suggested they might have slightly different properties. In fact, the ATCV-1 GMD does not form GDP-d-rhamnose, and so, it lacks the second enzyme activity [37]. Both GMD enzymes bound NADPH tightly, and this association was essential for the stabilization and function of both enzymes, even though NADP^+^ is the co-enzyme required to initiate the GMD catalytic cycle. Phylogenetic analyses established that the PBCV-1 GMD is the most evolutionarily diverged of all the GMDs, whereas the ATCV-1 GMD was in a clade of bacterial GMDs [37].

The GMER enzymes from PBCV-1 and ATCV-1 have 63% amino acid identity to each other and phylogenetically are more similar to one another and to other GMERs than are the two GMDs. The possible evolutionary consequences of these differences have been discussed previously [37]. Both fucose and rhamnose are constituents of the glycans attached to the PBCV-1 and ATCV-1 major capsid proteins (see below). However, the PBCV-1 glycan contains three rhamnose residues, with one in the D-configuration, whereas only one with L-configuration is present in the ATCV-1 glycan. Perhaps there was enough natural selection pressure on the PBCV-1 GMD gene to evolve to synthesize GDP-d-rhamnose, whereas the ATCV-1 GMD did not face this pressure.

ATCV-1 and all of the SAG viruses, however, encode another enzyme, UDP-d-glucose 4,6-dehydratase (UGD), that is one of two enzymes involved in the synthesis of L-rhamnose [38], and this enzyme may contribute to rhamnose synthesis. The PBCV-1 host chlorella, which is closely related to the ATCV-1 host chlorella, encodes the second enzyme in the rhamnose pathway [22], and so the host is predicted to be able to synthesize the rhamnose required for ATCV-1 glycan synthesis.

## 4. Unusual Attachment of Glycans to the Chlorovirus Major Capsid Proteins

Structural proteins of many viruses, such as rhabdoviruses, herpesviruses, poxviruses and paramyxoviruses, are glycosylated. Glycans contribute to the protease resistance and the antigenicity of these viruses. Most virus glycans are linked to Asn in the protein via *N*-acetylglucosamine, although some viruses also have *O*-linked glycans attached to either Ser or Thr residues via an amino sugar, usually *N*-acetylglucosamine or *N*-acetylgalactosamine. Typically, viruses use host-encoded glycosyltransferases and glycosidases located in the endoplasmic reticulum (ER) and Golgi apparatus to add and remove *N*-linked sugar residues from virus glycoproteins either co-translationally or shortly after translation of the protein. This post-translational processing aids in protein folding, progression in the secretory pathway and in the regulation of host-virus interactions [39,40,41,42]. After folding the protein, virus glycoproteins are transported by host-sorting and membrane-transport functions to virus-specified regions in host membranes where they displace host glycoproteins. Progeny viruses then bud through these virus-specific target membranes, which is usually the final step in the assembly of infectious viruses. Thus, nascent viruses only become infectious after budding through the membrane, usually the plasma membrane, as they exit the cell. Consequently, the glycan portion of virus glycoproteins is host-specific. The theme that emerges from these viruses is that virus glycoproteins are synthesized and glycosylated by the same processes as host glycoproteins. Therefore, the only way to alter virus protein glycosylation is to either grow the virus in a different host or have a mutation that alters the virus protein glycosylation site.

Unlike the process described above, glycosylation of the chlorovirus major capsid proteins differs from that scenario because the viruses encode most, if not all, of the machinery for the process. In addition, the process occurs in the cytoplasm. The conclusion that the chlorovirus PBCV-1 major capsid protein (Vp54, gene *a430l*) is glycosylated by a different mechanism than that used by other characterized viruses originally arose from antibody studies [43]. Rabbit polyclonal antiserum prepared against intact PBCV-1 particles inhibited virus plaque formation by agglutinating the virions. However, spontaneously-derived, antiserum-resistant, plaque-forming variants of PBCV-1 occurred at a frequency of 10^−5^–10^−6^. At the time of the 1993 publication, these antiserum-resistant variants fell into four serologically-distinct classes; two additional antigenic variants have subsequently been isolated for a total of six variants (Table 3). Polyclonal antisera prepared against members of each of these antigenic classes react predominately with the Vp54 equivalents from the viruses in the class used for the immunization. Each of the Vp54 proteins from the antigenic variants migrated faster on sodium dodecyl sulfate polyacrylamide gel electrophoresis (SDS-PAGE) than those of the strains from which they were derived, indicating a lower molecular weight. However, all of the de-glycosylated Vp54 proteins migrated at the same rate on SDS-PAGE, indicating that the differences resided in the size of the attached glycans. In addition, the nucleotide sequence of the *a430l* gene in each of the variants was identical to the wild-type gene, which verified that the polypeptide portion of Vp54 was not altered in the mutants. Western blot analyses of Vp54 proteins isolated from the variants, before and after removing the glycans with trifluoromethane-sulfonic acid or altering the glycan with periodic acid, also supported the notion that the antigenic variants reflected differences in the Vp54 glycans, not the Vp54 polypeptide [43].

All of the glycan antigenic variants form plaques on their *C. variabilis* NC64A host, so one can infer that the glycans are not directly involved in virus infection and virus replication. However, anecdotal evidence suggests that the glycans are important in virus stability because the variants with the smallest glycans do not remain infectious in storage as long as wild-type virus.

Additional observations supported the concept that PBCV-1 Vp54 glycosylation was unusual: (1) unlike viruses that acquire their glycoproteins(s) by budding through a plasma membrane, which results in infectious particles, plaque-forming PBCV-1 particles accumulate inside the host 30–40 min before virus release [47]; (2) all of the antigenic variants were grown in the same host so the glycan differences are not due to the host; (3) polyclonal antibodies to Vp54, the major capsid protein, do not react with host glycoproteins; (4) the Vp54 protein lacks an ER and Golgi signal peptide; (5) unlike most glycoproteins that exhibit size micro-heterogeneity, PBCV-1 Vp54 appears homogeneous on SDS-PAGE; in addition, mass spectrometry analysis only revealed one satellite peak that differed from the main peak by 140 Da, the approximate weight of either one arabinose or xylose residue [46]; and (6) the ability to easily crystallize Vp54 as a homotrimer provided additional evidence that the protein is essentially homogeneous [48,49].

**Figure 3:**
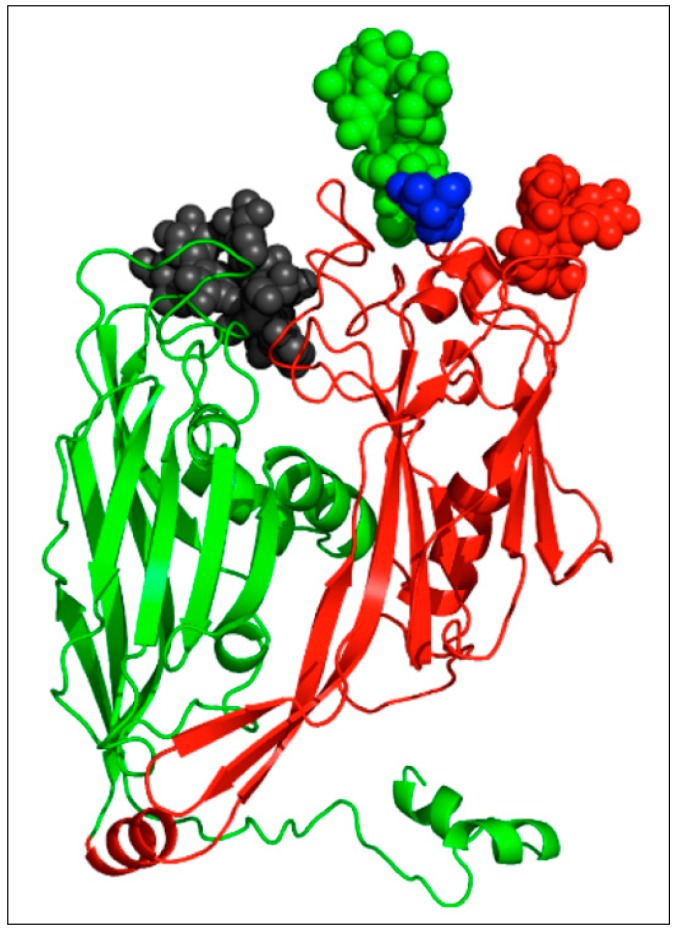
Structure of the revised PBCV-1 Vp54 monomer. The two jelly-roll domains are colored in green and red, respectively. The glycans located on the surface are shown as a space-filling representation of their atoms and are colored according to the residue they are attached to (Asn-280: green, Asn-302: black, Asn-399: red, Asn-406: blue). Taken from De Castro et al. [49] with permission.

Evidence that the *N*-linked Vp54 glycans are not attached to the Vp54 protein by a traditional *N*-linkage was initially obtained from the X-ray crystal structure of the protein. The structure revealed that the protein had four *N*-linked glycans at Asn positions 280, 302, 399 and 406 [48]. None of these Asn were located in an Asn-X-(Thr/Ser) sequon sequence commonly recognized by ER located glycosyltransferases [50,51,52]. This finding also explained why prior attempts to remove Vp54 glycans with enzymes that cleave traditional *N*-linked glycans were unsuccessful [53] Nandhagopal et al. [48] also reported that Vp54 contained two *O*-linked glycans. However, re-examination of the X-ray crystal data (Figure 3) indicates that no *O*-linked glycans are present in the protein [49], which agrees with our unsuccessful attempts to detect them by chemical procedures.

## 5. Glycan Structures Attached to Chlorovirus Major Capsid Proteins

**Figure 4:**
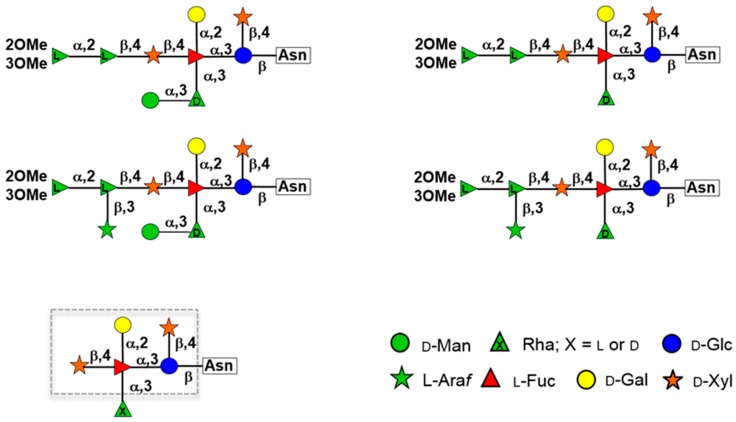
Structures of PBCV-1 Vp54 *N*-glycans. Arabinose and mannose are not stoichiometric substituents and create four different glycoforms. The two on the left are the most abundant, and both have mannose. The structure at the bottom represents the conserved core oligosaccharide that is present in all of the chloroviruses studied to date. Residues within the box are those strictly conserved, while rhamnose (outside the box) is a semi-conserved element because its absolute configuration is virus dependent. The figure was modified from De Castro et al. [34,54] with permission.

The structures of the PBCV-1 Vp54 *N*-linked glycans were reported recently, and they consist of 8–10 neutral monosaccharide residues, producing a total of four glycoforms (Figure 4) [54]. These structures do not resemble any structure previously reported in the three Domains of Life. Among their most distinctive features are: (1) the four glycoforms share a common core structure, and the four glycoforms are related to the non-stoichiometric presence of two monosaccharides, L-arabinose and D-mannose; the most abundant glycoform consists of nine neutral monosaccharide residues organized in a highly-branched fashion; (2) the glycans are attached to the protein by a β-glucose linkage, which is rare in nature and has only been reported in glycoproteins from a few organisms [55,56,57,58]; and (3) the glycoform contains a dimethylated rhamnose as the capping residue of the main chain, a hyper-branched fucose residue and two rhamnose residues with opposite absolute configurations.

Attempts to fit the Vp54 glycan structures into the original Vp54 X-ray crystal structure [48] were unsuccessful and led to a re-examination of the original structure. This re-examination produced a structure that was compatible with the four *N*-linked glycan structures (Figure 3) [49]. As mentioned above, the revised structure lacks the two *O*-linked glycans reported originally.

The PBCV-1 Vp54 has a molecular weight of 53,790 Da. The *a430l* gene encodes Vp54 with a predicted molecular weight of 48,165 Da so the combined sugars have a molecular weight of 5625 Da, which is about the weight of the four glycans. Vp54 was also reported to have a myristic acid attached to the carboxyl portion of the protein [59]. However, myristic acid has not been observed in any of the recent Vp54 structural experiments, and so, its status is currently unknown. The structures of the Vp54 glycans from the PBCV-1 antigenic variants, referred to above, are currently being determined, and as expected, the structures are truncated forms of the wild-type PBCV-1 glycans [44]. PBCV-1 particles were reported to have two additional glycoproteins in addition to Vp54 [59]. Both of these glycoproteins react with the PBCV-1 antibody, and so, the glycan structures are predicted to be similar or identical to the glycans associated with Vp54. The gene encoding one of these proteins (Vp260) was identified (gene *a122r*). Gene *a122r* homologs are common in the chloroviruses, and some of the viruses have as many as five copies of the gene [60]. The role that Vp260 plays in the PBCV-1 virion is unknown. 

**Figure 5:**
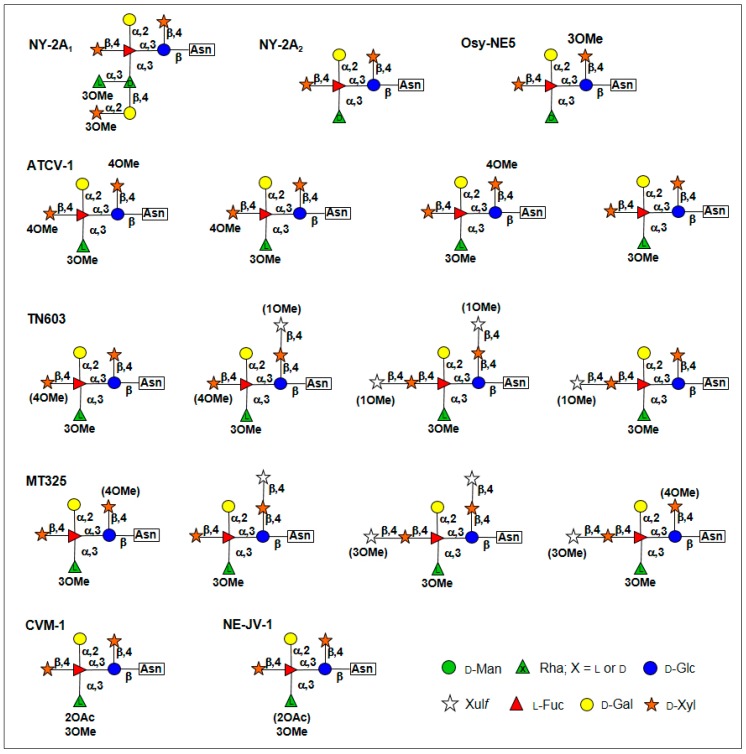
Structures of *N*-glycans from seven chloroviruses representing all four chlorovirus types. Substituents in brackets are not stoichiometric. All sugars are in the pyranose form, except where specified. Virus NY-2A is an NC64A virus, virus Osy-NE5 an Osy virus, viruses ATCV-1 and TN603 SAG viruses and MT325, CVM-1 and NE-JV-1 Pbi viruses. This figure was modified from [6,34,35] with permission.

The glycan structures of the major capsid proteins from seven more chloroviruses, which represent all four chlorovirus types, were recently reported (Figure 5) [6,34,35]; collectively, all of the glycans have a common core region (outlined in Figure 4). The common core region consists of a pentasaccharide with a β-glucose linked to an Asn residue, which is not located in the typical sequon Asn-X-(Thr/Ser). The glucose has a terminal xylose unit and a hyperbranched fucose, which is in turn substituted with a terminal galactose and a second xylose residue. The third position of the fucose unit is always linked to a rhamnose, which is a semi-conserved element because its configuration is virus dependent. Additional decorations occur on this core *N*-glycan and represent a molecular signature for each chlorovirus.

## 6. Chlorovirus PBCV-1 Encoded Glycosyltransferases

In addition to the two glycosyltransferases, hyaluronan synthase and chitin synthase previously described, the 43 chloroviruses collectively encode eight putative glycosyltransferases (Table 4). Six of these eight glycosyltransferase-encoding genes are in PBCV-1; they are scattered throughout the PBCV-1 genome. None of these six PBCV-1 encoded glycosyltransferases have an identifiable signal peptide that would target them to the ER. Furthermore, with the exception of PBCV-1 glycosyltransferases A473L (six transmembrane domains; CESA CelA-like) and A219/222/226R (nine transmembrane domains; CXCX-2), none of the four remaining PBCV-1 encoded glycosyltransferases are predicted to have transmembrane domains. Therefore, these enzymes are expected to be soluble proteins. The genes for the six PBCV-1 encoded glycosyltransferases are expressed early during PBCV-1 infection [61]. Thus, assuming the enzymes are stable, they would be available for adding sugars to the Vp54 glycans during virus replication.

The PBCV-1 *a064r* gene encodes a 638-amino acid protein with three predicted domains. The N-terminal 211 amino acid domain resembles a “fringe-class” of glycosyltransferases (GT-GTA) and contains the last four of the five conserved motifs characteristic of this group of glycosyltransferases [62,63], including the proposed catalytic amino acids, the Asp-X-Asp sequence in motif 3 and the first Asp residue in motif 5. However, spacing between some of the four motifs differs from that of the fringe-glycosyltransferases. As mentioned above, the A064R protein, which is only present in five NC64A viruses, lacks both an identifiable signal peptide that would target the protein to the ER and a membrane-spanning motif, in contrast to “fringe” glycosyltransferases.

The 211-amino acid A064R glycosyltransferase domain was cloned, and the recombinant protein was crystallized [64]. The 1.6 Å crystal structure of the peptide has a mixed α/β fold containing a central, six-stranded β sheet flanked by α helices. The overall fold is similar to the catalytic domains in retaining glycosyltransferases in the GT-A group, family 34, although the amino acid similarity between them is low. Zhang et al. [64] suggested that the A064R glycosyltransferase bound to UDP-glucose better than to UDP-galactose or UDP-*N*-acetyl glucosamine. However, these binding experiments were conducted prior to knowing the Vp54 glycan structures. Now, there is evidence that the glycosyltransferase domain adds L-rhamnose to the distal xylose residue in the core structure [45].

Analysis of the six PBCV-1 antigenic variants revealed mutations in *a064r* that correlated with a specific antigenic class, B (EPA-1) (Table 3). The *a064r* gene in all six of these antigenic variants was sequenced to determine if mutations in *a064r* correlated with the EPA-1 antigenic variation [46]. The *a064r* sequences from three of the mutants had single nucleotide substitutions, which produced a single amino acid substitution in the glycosyltransferase portion of the A064R protein. Two of the amino acid substitutions occurred in the Asp-X-Asp motif (domain 3), and the other one was in domain 4. A fourth variant had an extra base in the coding sequence, which created a frame shift mutation in the gene. Finally, the entire gene was deleted in the other two antigenic variants.

Dual infection experiments with some of the different antigenic variants established that viruses containing wild-type *a064r* complemented and recombined with viruses that contained variant *a064r* to form wild-type virus. Therefore, it was concluded that *a064r* encodes a glycosyltransferase involved in the synthesis of the Vp54 glycan [46].

As noted above, the protein product of the *a064r* gene contains three domains with domain 1 being the glycosyltransferase. Domain 2 does not match anything in GenBank, but the C-terminal domain 3 is predicted to be a methyltransferase. We suspect that this C-terminal domain of approximately 200 amino acids is involved in methylating the terminal L-rhamnose in the Vp54 glycan [45].

A homolog of PBCV-1 glycosyltransferase, A546L (GT-GT4), has also been produced and crystallized [65]. The *a546l* gene homolog was from another NC64A chlorovirus NY-2A (gene *b736l*), and the 396-amino acid protein resembles members in the GT4 family of glycosyltransferases in the CAZy classification [66,67]. However, its biochemical function remains to be elucidated.

Of the eight glycosyltransferases encoded by the 43 chloroviruses, only two of them, homologs of PBCV-1 A111/114R and A075L, are present in all of the viruses, and so, they are predicted to be involved in the synthesis of the core glycan structure. A111/114R is especially interesting because it is predicted to have at least two glycosyltransferase catalytic domains.

Now that structures of the glycans from the chlorovirus major capsid proteins are becoming available, one can begin to characterize the viral encoded glycosyltransferases biochemically. One question that needs to be addressed is: Are the sugars added sequentially to the Vp54 protein backbone or are the glycans initially synthesized independently of Vp54, possibly on a lipid carrier and then attached to the protein in a single step? A slight variation of these two possibilities is that the core glycan is synthesized independently of the protein and then attached to Vp54. Additional sugars could then be added sequentially to these core glycans [68]. We suspect that this viral encoded glycosylation pathway represents a previously undescribed pathway, possible even a pathway that existed in eukaryotes prior to the ER and Golgi glycosylation pathway [18].

## 7. Additional Chlorovirus Encoded Sugar Metabolism Enzymes

Besides the chlorovirus-encoded enzymes described above, the viruses have four additional genes predicted to encode enzymes involved in sugar metabolism (Table 2). Recombinant proteins have not been produced from any of these genes, and so, it is unknown if they encode functional enzymes. These putative enzymes include an acetyltransferase (AT) encoded by all 43 chloroviruses, a D-lactate dehydrogenase (D-LD) encoded by 32 chloroviruses, fumarate reductase (FRD) encoded by five chloroviruses and ADP-ribosyl glycohydrolase (ADP-RGH) encoded by nine chloroviruses, all but two of which are Pbi viruses. The roles these putative enzymes play in the viral life cycles are unknown.

## 8. Chlorovirus-Encoded Polysaccharide Degrading Enzymes

In addition to the polysaccharide synthesizing enzymes described above, the chloroviruses also encode polysaccharide-degrading enzymes (Table 5). The chloroviruses are unique among viruses infecting eukaryotic organisms in that they, like bacteriophages, need to penetrate a rigid algal cell wall to initiate infection. The icosahedral shaped chlorovirus PBCV-1 has a spike-like structure at one vertex [13], which appears to make the initial contact with the cell wall of its host, *C. variabilis* NC64A [69]. Attachment is immediately followed by host cell wall degradation at the point of contact by a virus-packaged enzyme(s) [70]. After wall degradation, the viral internal membrane fuses with the host membrane to produce a narrow (~5 nm in inner diameter), membrane-lined tunnel, which allows entry of the viral DNA and some viral proteins [71]. This membrane fusion results in immediate host membrane depolarization [72] and potassium ion efflux [73]. This process results in an empty capsid remaining on the host cell surface.

In addition to virus entry into the host cells, nascent infectious PBCV-1 viruses exit the cells at 6–8 h PI by lysis of the plasma membrane and the cell wall. Therefore, it is not surprising that the chloroviruses encode polysaccharide-degrading enzymes in order to enter and exit the host cell. In fact, PBCV-1 encodes five such enzymes (Table 5), including two chitinases [74,75], a chitosanase [74,76], a β-1,3 glucanase [77] and an alkaline alginate lyase [78] or a polysaccharide lyase, cleaving chains of β- or α-1,4-linked glucuronic acids [79,80]. Recombinant proteins have been produced from each of these genes and shown to have the expected activity. Interestingly, the β-1,3 glucanase gene is expressed very early and disappears by 60 min PI. The protein is also made very early and disappears by 90 min PI [77]. Therefore, this enzyme is unlikely to be involved in either viral entry or viral exit from the cell. One possible function for the enzyme is to degrade host β-1,3 glycans, which might serve as host storage polysaccharides. Gene transcripts from the other four polysaccharide-degrading enzymes are present throughout the viral life cycles [74,81].

Experiments conducted about 30 years ago established that a crude enzyme preparation made from PBCV-1 lysates, named lysin, had good wall degrading activity and could be used to produce *C. variabilis* NC64A protoplasts [82,83]. Therefore, it was assumed that one or more of the five PBCV-1 encoded enzymes would be packaged in the PBCV-1 virion and be responsible for degrading the host cell wall at the point of infection. In fact, Yamada et al. [76] reported that a chitosanase activity was packaged in a closely-related chlorovirus, CVK2. However, an ensuing report [75] indicated that the CVK2 chitosanase activity was due to incomplete purification of the virion. Subsequently, a PBCV-1 proteome study identified 148 virus-encoded proteins and one host-encoded protein in highly purified virions [10]. Surprisingly, none of the five polysaccharide-degrading enzymes were packaged in the PBCV-1 virions.

Consequently, the 148 virus-encoded proteins packaged in the PBCV-1 particles were re-examined for possible polysaccharide or cell wall degrading activity. This effort revealed that one of the PBCV-1-encoded proteins packaged in the virion, A561L, has a putative glycosyl hydrolase domain. A recombinant protein produced from this domain has cell wall degrading activity, and the protein is under active investigation [84]. Homologs of the A561L domain (named A561L lysin) are present in most of the chloroviruses (Table 5), but not all. For example, viruses NYs-1 and CVR-1 appear to lack an *a561l* gene homolog encoding this domain, and the similarity between the predicted A561L homolog from viruses NY-2B and WI0606 is not very high. The apparent absence of the protein from these viruses deserves to be investigated further because one would expect the enzyme(s) that degrades the host cell wall during virus infection would be highly conserved.

Twenty-four of the 43 chloroviruses encode a protein that has a polysaccharide deacetylase domain (Table 5). Viruses in three of the four types (Osy being the exception) have the gene, but it is also missing in some viruses in each of the three types, so the gene is clearly not required for the success of the viruses. Its role might be to remove the acetyl group from chitin during host cell wall degradation. 

In addition to these glycolytic enzymes, 42 of the 43 chloroviruses, encode a functional glycosylase protein that initiates pyrimidine photodimer excision [85,86]. The enzyme is part of a DNA repair pathway.

## 9. Conservation of the Chlorovirus Encoded Sugar Enzymes

Only three of the 21 chlorovirus encoded proteins listed in Table 1, Table 2 and Table 4 are present in all 43 chloroviruses, and these would be considered to be core proteins. The three are an acetyltransferase (AT), an exostosin glycosyltransferase (EXT) and a family A glycosyltransferase (GT-A). As noted above, we predict that the two glycosyltransferases are involved in the synthesis of the glycan core attached to the major capsid protein. The predicted function of the acetyltransferase is unknown.

Two of the seven viral encoded proteins involved in polysaccharide degrading activity are conserved in all of the viruses, the chitosanase (CHIS) and a putative bifunctional chitinase/lysozyme (BCHIL) (Table 4). Presumably these two enzymes play a role in the release of the nascent viruses from the cell. As indicated above, they are not packaged in the PBCV-1 virion, and so, they are not involved in the immediate early virus infection process.

The presence or absence of some of the chlorovirus sugar encoding enzymes displays some interesting patterns. For example, the GMD and GMER encoding genes are present in all of the NC64A, SAG and Osy viruses and absent in all of the Pbi viruses. This observation would suggest that the three virus types that have these genes would be more closely related to each other than to the Pbi viruses. However, a phylogenetic tree that shows the evolutionary relationship between the 43 viruses based on 29 concatenated core proteins [6] indicates that SAG and Pbi viruses are in the same branch and that the NC64A and Osy viruses are in a separate branch. Therefore, these results would imply that the SAG and NC64A/Osy viruses either acquired the genes separately after the four virus types had separated from a common ancestor or that the chlorovirus ancestor had both genes and for some reason, they were lost in the Pbi lineage.

Most of the other protein patterns are even more difficult to explain. For example, the GFAT encoding gene is present in all of the Pbi viruses and present in 11 of the 14 NC64A viruses and one SAG virus. Several of the other genes have similar complicated patterns and await explanations.

## 10. Sugar Enzymes Coded by Other Large DNA Viruses

This review has focused on carbohydrate enzymes encoded by the chloroviruses, primarily because these enzymes have been the most intensively studied. However, as new giant viruses are being discovered and their genomes sequenced, it is clear that some of them encode putative enzymes involved in carbohydrate manipulations. The most extensively studied of these other large DNA viruses is *Acanthamoeba polyphaga* mimivirus, which has genes encoding both glycosyltransferases and nucleotide sugars (see the recent review by Piacente et al. [87]). Other large DNA viruses encoding putative sugar manipulating enzymes include prasinoviruses (family *Phycodnaviridae* like the chloroviruses) that infect small marine green algae, including *Ostreococcus*, *Bathycoccus* and *Micromonas* species; these viruses have clusters of putative genes for enzymes involved in nucleotide-sugar metabolism and glycosyltransferases [88]. Similar genes are present in other members of the *Mimiviridae* family, including *Phaeocystis globosa* virus [89] and *Cafeteria roenbergensis* virus [90]. Putative glycosyltransferase-encoding genes have also been reported in the genomes of pandoraviruses [91], *Pithovirus sibericus* [92] and *Mollivirus sibericus* [93].

In conclusion, it is becoming clear that virus-encoded sugar-manipulating enzymes and glycosylation systems can no longer be considered a hallmark solely of cellular organisms, but that some viruses also encode unique and complex glycan systems, which are still largely unknown. One encourages young glycobiologists to consider working on some of these systems.

## Figures and Tables

**Table 1:** Chlorovirus encoded enzymes involved in the synthesis of polysaccharides.

Host	Viruses	HAS ^1^	CHS ^2^	CBP ^3^
**NC64A**	AN69C		390R	395L, 438L
	AR158		C418R	C423L, C475L
	CviK1	102R	365R	370L, 414L
	CvsA1		375R	380L, 427L
	IL-3A			386L, 432L
	IL-5-2s1	134L		503L, 562L
	KS-1B			314L, 360L
	MA-1D	485L	362R	367L, 472L
	MA-1E		113L	407L, 451L
	NE-JV-4			390L
	NY-2B			542L, 484L
	NY2A		B139R, B472R	B480L
	NYs-1	137R		360R, 495L, 555L
	PBCV-1	**^a^ A098R**		A333L, A348Dl, A383R
**SYN**	OSY-NE-5	038R		167L, 184L
**Pbi**	AP110A		152L, 175R	828R
	CVA-1		150L, 169R	834R
	CVB-1		177L	791R
	CVG-1	146R		792R
	CVM-1		165L, 186R	832R
	CVR-1			838R
	CZ-2			798R
	Can18-4	163R		839R
	FR483	N124R		N690R
	FR5L		151L	797R
	MT325	M128R		M701R
	NE-JV-1		278R, 282R	734L
	NW665	133R		821R
	OR0704.2.2	116R		804R
**SAG**	ATCV-1			Z734R
	Br0604L		834R	431R
	Can0610SP			438R, 442R
	Canal-1		746R	405R
	GM0701		852R	436R
	MN0810		087R, 900R	466R, 468R, 531L
	MO0605SPH			435R
	NE-JV-2			462R
	NE-jv-3			431R
	NTS-1		893R	461R, 463R, 529L
	OR0704.3			431R
	TN603		869R	425R
	W10606			457R

^1^ Hyaluronan synthase; ^2^ chitin synthase; ^3^ chitin binding proteins, except for the chitinase proteins reported in Table 5; ^a^ the recombinant protein has the predicted activity. The numbers refer to the protein names, and the R and L refer to the strand orientation.

**Table 2:** Chlorovirus encoded enzymes involved in sugar metabolism.

Host	Viruses	GFAT ^1^	UDP-GlcDH ^2^	GMD ^3^	GMER ^4^	UGD ^5^	AT ^6^	D-LD ^7^	ADP-RGH ^8^	FRD ^9^
**NC64A**	AN69C	109R	384R, 487R	129R	334L		739L	055R		
	AR158	C132R	C413R, C729L	C155R	C344L		C767L			
	CviK1	105R	359R, 662L	122R	312L		742L	055R		
	CvsA1	064R	368R	083R	321L		716L	131R		
	IL-3A	104R	375R, 685L	126R	326L		726L	051R		
	IL-5-2s1	130R	492R, 858L	106L	417L		896L			
	KS-1B			066R	261L		643L			
	MA-1D	481L	355R, 838L	456L	284L		872L			
	MA-1E	116R	396R	134R	355L		806L	045R		
	NE-JV-4			131R	340L		740L	064R		
	NY-2B		473R, 836L	185R	408L		881L			
	NY2A	B143R	B465R	B163R	B395L		B853L			
	NYs-1	143R	483R, 846L	167R	404L		879L			
	PBCV-1	**^a^** **A100R**	**^a^** **A609L**	**^a^** **A118R**	**^a^** **A295L**		A654L	A053R		
**SYN**	OSY-NE-5	039R		045R	139L		340L	015R	308R	
**Pbi**	AP110A	071R	146L				893R	053L		
	CVA-1	056R	144L				900R	040L	205R	
	CVB-1	071R	172L				856R	056L	221R	
	CVG-1	050R	812L				857R			
	CVM-1	069R	159L				893R	052L	222R	
	CVR-1	062R	151L				906R	046L	210R	
	CZ-2	059R	718L				865R	048L		917L
	Can18-4	061R	859L				908R	048L	212R	
	FR483	N035R	N712L				N747R		N170R	
	FR5L	087R	145L				863R	076L		
	MT325	M036R, M037R	M719L				M758R	M026L		
	NE-JV-1	081R	291R				861R	810L		
	NW665	046R	846L				889R		189R	
	OR0704.2.2	062R	722L				862R	051L		
**SAG**	ATCV-1		Z571L	**^a^** **Z804L**	Z282L	**Z544R**	Z147L	Z295L		
	Br0604L		667L, 839R	934L	332L	631R	173L	350L		
	Can0610SP		687L	965L	338L	658R	170L	355L		
	Canal-1		605L, 751R	847L	329L	576R	188L	343L	898L	886R
	GM0701		664L, 856R	954L	337L	629R	180L	354L		
	MN0810	887L	720L, 904R	992L	365L	689R	204L	379L		
	MO0605SPH		656L	897L	341L	625R	181L	359L		943R
	NE-JV-2		708L	981L	367L	672R	186L	383L		
	NE-JV-3		679L	935L	332L	648R	175L	347L		981R
	NTS-1		714L, 898R	1012L	378L	681R	188L	391L		
	OR0704.3		676L	960L	335L	639R	179L	354L		
	TN603		659L, 873R	966L	326L	625R	179L	342L		
	W10606		679L	916L	360L	651R	185L	375L		962R

^1^ Glutamine-fructose-6-phosphate aminotransferase; ^2^ UDP-glucose-6-dehydrogenase; ^3^ GDP-d-mannose dehydratase; ^4^ GDP-4-keto-6-deoxy-d-mannose epimerase/reductase (=GDP-l-fucose synthase 2); ^5^ UDP-d-glucose 4,6-dehydratase, ^6^ acetyltransferase; ^7^ d-lactate dehydrogenase; ^8^ ADP-ribosylglycohydrolase; ^9^ fumarate reductase; ^a^ the recombinant proteins have the predicted activities. The numbers refer to the protein names, and the R and L refer to the strand orientation.

**Table 3:** PBCV-1 antigenic variants that affect the molecular weight of the major capsid glycoprotein.

Antisera Classes		Predicted MW (kDa) ^c^	SDS-PAGE Estimates (kDa) ^d^
Class ^a^	Label ^b^		
+	Wild-type	54.1	54
F	CME6	54	not determined
A	P91	52.8	53
E	EPA-15	52.8	not determined
B	EPA-2	51.6	52
C	E1L3	51.1	51
D	P1L6	50.5	50.5

^a^ Listed in order of predicted molecular weight based on nuclear magnetic resonance (NMR) analysis (De Castro et al., [44,45]); ^b^ representative mutant strain label; ^c^ the gene encoding the PBCV-1 major capsid protein (*a430l*) is wild-type in sequence and does not vary among antisera classes; ^d^ Graves et al. [46]. MW, molecular weight.

**Table 4:** Chlorovirus encoded enzymes involved in synthesizing glycans attached to virus major capsid proteins.

Host	Viruses	EXT ^1^	GT-A ^2^	GT-GT4 ^3^	GT-GTA ^4^	CESA CelA-Like ^5^	CSCS-2 ^6^	GT ^7^	GT17 ^8^
**NC64A**	AN69C	078L	123R	559R	065R	104R	255R		
	AR158	C093L	C150R	C661L			C265R		C559R
	CviK1	080L	117R	594L		518L	242R		
	CvsA1	039L	077R	611L		535L	247R		
	IL-3A	071L	120R	606L	060R	099R	240R		
	IL-5-2s1	175R	109L	773L			313R		649R
	KS-1B	024L	060R	528L	009R	046L	170R		
	MA-1D	531R	4549L	753L			194R		637R
	MA-1E	533R	128R	702L		626L	281R, 210R		
	NE-JV-4	085L	124R	631L	074R	108L	250R		
	NY-2B	116L	180R	754L		160R	323R		633R
	NY2A	107L	B159R	B736L					B618R
	NYs-1	098L	162R	760L			187R		641R
	PBCV-1	A075L	A111/114R	A546L	A064R	A473L	A219/222/226R		
**SYN**	OSY-NE-5	025L	044R	283L			097L		
**Pbi**	AP110A	013L	548R			226R		970R	
	CVA-1	016L	532R			220R		971R	
	CVB-1	025L	538R			232R	811L	918R	
	CVG-1	019L	520R			217R	815L	920R	
	CVM-1	022L	550R			237R		953R	
	CVR-1	020L	545R			225R		977R	
	CZ-2	012L	532R	380L			822L	932R	
	Can18-4	020L	557R			229R	862L	971R	
	FR483	N012L	N472R			N191R	N715L	N805R	
	FR5L	046L	537R				819L	926R	
	MT325	M009L	M467R			M186R	M721L	M813R	
	NE-JV-1	079L	464L				801R	930R	
	NW665	015L	532R				849L	955R	
	OR0704.2.2	017L	549R	382L			823L	923R	
**SAG**	ATCV-1	Z830R	Z120R	Z667L		Z178L, Z823R, Z417L	Z425R	Z347R	
	Br0604L	959R	137R			225L, 952R, 483L	489R	399R	
	Can0610SP	1007R	140R	789L		210L, 1002R, 978L, 487L	495R	407R	
	Canal-1	874R	141R	164L		871R	447R	380R	
	GM0701	977R	141R	752L		228L, 975R, 486L	493R	405R	
	MN0810	1009R	165R			244L		424R	
	MO0605SPH	932R	138R	164L		230L, 926R, 479L	488R	412R	
	NE-JV-2	1020R	150R	804L		1015R, 992L, 516L	523R	438R	
	NE-JV-3	970R	145R	778L		215L, 210L, 964R, 484L	493R	407R	
	NTS-1	1044R	156R			1016L, 516L		441R	
	OR0704.3	1006R	146R	944L		1001R, 972L, 485L, 219L	493R	404R	
	TN603	991R	141R			226L, 986R, 475L	483R	400R	
	WI0606	951R	141R			233L, 945R, 506L	514R	430R	

^1^ Exotosin glycosyltransferase; ^2^ glycosyltransferase family A; ^3^ glycosyltransferase GT4-type super family; ^4^ glycosyltransferase GTA-type super family; ^5^ CESA CelA-like cellulose synthase catalytic subunit (UDP-glucose as substrate); ^6^ cellulose synthase catalytic subunit; ^7^ glycosyltransferase; ^8^ glycosyltransferase family 17.

**Table 5:** Chlorovirus encoded enzymes involved in degrading polysaccharides.

Host	Viruses	CHI ^1^	CHIS ^2^	GUN ^3^	BCHIL ^4^	Lysin ^5^	GH ^6^	CD ^7^	ALGL ^8^
**NC64A**	AN69C	297R	331L	102L	204R	540R		387L	250L
	AR158		C342L	C126L	C220R	C681L		C415L	C263L
	CviK1		309L	99L	194R	610L		362L	237L
	CvsA1		318L	058L	200R	627L		371L, 373L	243L
	IL-3A	285R	323L	097L	196R	624L		378L	235L
	IL-5-2s1	373R	415L	140R	262R	796L		495L	310L
	KS-1B	211R	257L	048R	132R	546L			166L
	MA-1D	241R, 242R	283L	491R	147R	777L		359L	191L
	MA-1E		352L	114R	194R, 229R	717L		399L	277L
	NE-JV-4	298R	337L	110R	205R	648L			246L
	NY-2B	367R	406L	158L	270R	777L		476L	319L
	NY2A		B393L	B137L	B239R	B756L		B469L	B288L
	NYs-1	360R	403L	134L	251R			487L	
	PBCV-1	**^a^ A260R**	**^a^ A292L**	**^a^ A094L**	**^a^ A181/182R**	**^a^ A561L**			**^a^ A215L**
**SYN**	OSY-NE-5	119R	138L	037L	117L	290L			099R
**Pbi**	AP110A	106R	122R	174L	942R	306R		158L	338R
	CVA-1	102R	114R	167L	942R	297R		156L	327R
	CVB-1	129R	143R		890R	310R		183L	342R
	CVG-1	105R	113R	142L	896R	303R			329R
	CVM-1	122R	133R	184L	927R	330R		171L	360R
	CVR-1	109R	121R	174L	948R			163L	335R
	CZ-2	070R	079R	114L	902R	263R			293R
	Can18-4	113R	121R	160L	944R	316R			349R
	FR483		N087R	N119L	N779R	N262R			N293R
	FR5L	098R	106R		897R	300R		157L	336R
	MT325	M085R	M091R	M124L	M791R	M258R			M289R
	NE-JV-1	328L	218L	275L	088R	592L	050L	269L	472L
	NW665		088R	128L	921R	281R			315R
	OR0704.2.2	074R	082R	113L	895R	257R			292R
**SAG**	ATCV-1	Z780L	Z204R	Z819L	Z814L	Z511L			Z771L
	Br0604L		253R, 254R	950L	902R, 942L	518L		832L	895L
	Can0610SP		244R	985R, 1001L	936R, 996R	613L			919L
	Canal-1	815L	242R	866L	138L, 855L	546L		743L	806L
	GM0701		251R	972L	917R, 963L	526L		846L	910L
	MN0810		271R	082L	162L, 1003L	659L		896L	959L
	MO0605SPH	871L	257R	920L	911L	588L			862L
	NE-JV-2	950L	258R	1000R, 1013L	1008R	634L			932L
	NE-JV-3	907L	244R	957L	948L	603L			897L
	NTS-1	973L	276R	1024R	1039R	556L		886L	961L
	OR0704.3		251R	979R, 999L	918R, 994R	604L			910L
	TN603		253R	983L	939R, 977L	520L		867L	931L
	WI0606	889L	262R	938L	929L	612L			878L

^1^ Chitinase; ^2^ chitosanase; ^3^ 1-3-beta glucanase; ^4^ bifunctional chitinase/lysozyme; ^5^ lysin homologs from encoded by PBCV-1 CDS *A561L*; ^6^ glycosyl hydrolase; ^7^ chitin deacetylase; ^8^ alanine alginate lyase; ^a^ the recombinant proteins have the predicted activity.
